# Epidemiology, Detection and Treatment of Malaria

**DOI:** 10.3390/tropicalmed9100235

**Published:** 2024-10-09

**Authors:** Wenn-Chyau Lee, Yee-Ling Lau

**Affiliations:** 1 Department of Parasitology, Faculty of Medicine, Universiti Malaya, Kuala Lumpur 50603, Malaysia; 2 A*STAR Infectious Diseases Labs (A*STAR ID Labs), Agency for Science, Technology and Research (A*STAR), 8A Biomedical Grove, Immunos #05-13, Singapore 138648, Singapore

## 1. Introduction

Malaria, one of the oldest infections to affect humans, incurs significant healthcare burdens across various parts of the world. This vector-borne disease is caused by *Plasmodium*, an apicomplexan parasite. Despite countless efforts to clear this disease from humans, nearly half of the world’s population still lives in malaria transmission risk areas, with approximately 70% of the malaria burden concentrated in 11 nations [1]. In recent years, the emergence and spread of *Plasmodium falciparum* strains that are resistant to artemisinin and its derivatives (which are the current first-line treatment regime) is one of the biggest challenges encountered in terms of control and elimination [2,3]. Notably, the parasites have evolved different strategies to resist artemisinin [4,5,6,7,8,9,10,11,12,13,14,15,16,17], complicating efforts to monitor their anti-malarial treatment sensitivity profiles. Drug resistance is driven by genetic mutations and adaptive mechanisms that allow the parasites to survive in the presence of the drug. As a result, accurately assessing the parasite’s sensitivity profile to antimalarials has become increasingly challenging, requiring more sophisticated techniques and a deeper understanding of the underlying drug resistance mechanisms. The situation is compounded by the emergence and spread of potentially fatal zoonotic malaria caused by *P. knowlesi* in Southeast Asia [18,19,20,21]. In addition, the currently available detection methods are not effective enough for accurate species identification and malaria surveillance, particularly in areas with mixed endemicity of human malaria parasites and simian malaria parasites, as well as places with asymptomatic cases harbouring parasite density of a sub-microscopic level [18,22,23,24,25,26]. As a result, works related to malaria surveillance and epidemiological studies are more challenging to undertake in these areas, especially when endemic areas are reduced to sporadic, isolated geographical pockets across a country or region. This also hampers a thorough deciphering of transmission dynamics specific to these locations, particularly the interactions of *Plasmodium* with its vectors, intermediate hosts, and the environment, which vary greatly depending on geography and the species of parasites involved [27,28,29,30,31]. This Special Issue compiles some of the latest updates on the epidemiology, diagnosis, and treatment of malaria.

## 2. An Overview of the Published Articles

Concerning the epidemiology of malaria, Melebari et al. (Contribution 1) described the influence of international travel on the dynamics of malaria burden in Saudi Arabia. Every year, Saudi Arabia receives millions of travellers from various parts of the world, particularly during Muslim pilgrimages. This study may serve as an important reference for countries that experience high influxes of foreign visitors, and those where the healthcare workers of these countries must be adequately prepared to handle diseases that are not commonly encountered in their local settings. Meanwhile, Garcia et al. (Contribution 2) highlighted the value of probabilistic data linkage across different health information systems within a country to recover important epidemiologic data that can be used for malaria burden surveillance. Having multiple independent databases with different management systems within a country will result in fragmented information, making it difficult to accurately decipher the actual epidemiologic landscape of an infection in a country. Reliable record linkages significantly improve the efficacy of extracting key information that can be used for disease surveillance. In this Special Issue, Quattara et al. (Contribution 3) contributed to the field of malaria epidemiology research by revealing the differences in malaria transmission dynamics between pre-school and school-age children in Southwestern Burkina Faso. In many healthcare settings, paediatric cohorts may have wide age ranges, and the different social activities that children of different age groups engage in may be overlooked. As school-age children leave their homes for school, they are more likely to be exposed to other environmental factors than pre-school children, resulting in different disease acquisition risks. This study highlights the need for specific and effective interventions for children of different age groups to further reduce the malaria burden in malaria-endemic areas. In view of this, it is highly recommended that the WHO’s conditional guidelines for intermittent preventive treatment in school-aged children living in regions with moderate to high malaria transmission be implemented.

Besides epidemiology, several works also investigated parasite–host interactions. Biabi et al. (Contribution 4) reported on the relationship between the polyclonality of *Plasmodium falciparum* within a human host and the host-derived antibody responses; polyclonality and parasitemia were negatively correlated with the level of IgG against some of the *P. falciparum*-derived antigens, such as MSP-1p19, MSP-3, and EBA175. Notably, this study also found no significant correlation between the frequency of polyclonal infection and the clinical manifestation of malaria. In a separate study, Noordin et al. (Contribution 5) highlighted the negative selection of the *P. knowlesi* MSP-1 (*pkmsp-1*) gene within the human host, reflecting the interactions between the simian parasite and its new non-natural intermediate host as part of the process of host adaptation. The interaction between *Plasmodium* and *Anopheles* is another important aspect of the parasite–host relationship that warrants further research attention. Liu et al. (Contribution 6) reported on the inhibitory effect of human defensin 5 on the development of *Plasmodium yoelii* within *Anopheles stephensi*. Importantly, such an inhibitory effect was mediated by human-derived peptides via the activation of the mosquito’s innate immunity. These findings may open the door to exploring new approaches to block the transmission of malaria parasites.

This Special Issue also received contributions covering aspects relating to diagnosis. Via a systematic review, Yalley et al. (Contribution 7) addressed the need for modern techniques to enhance the speed and efficacy of malaria diagnosis in low and lower-middle-income countries, which are significantly affected by malaria. Hence, there is a pressing need to increase financial support and initiatives to assist these nations in improving their malaria diagnosis efforts, which is pivotal for the overall effort to eliminate malaria. Indeed, Lai et al. (Contribution 8) presented an innovative molecular detection technique that allows rapid and accurate malaria diagnosis to be carried out even in areas with less-equipped diagnosis facilities. Of note, the technique described by the team can be used to detect the potentially fatal zoonotic malaria caused by *P. knowlesi*, which is of increasing prevalence in Southeast Asia. Due to its shared morphological characteristics with human malarial parasites, the light microscopy-based identification of malaria parasites at the species level has become more challenging. Furthermore, none of the rapid diagnostic test kits currently available can be used to reliably and specifically detect *P. knowlesi*. The findings from this study may facilitate the development of a reliable and practical diagnostic tool covering both human and zoonotic malaria.

Finally, for the treatment of malaria, a review contributed by Zheng et al. (Contribution 9) offers insights into drug resistance development in malarial parasites and discusses the initiatives to address this challenge, contributing to the broader effort to eradicate malaria from the human population. Indeed, the spread of multi-drug-resistant malaria parasites has become a pressing issue for many authorities battling against the transmission of malaria. Rapid, systematic, and effective measures must be taken to mitigate this issue. Otherwise, decades of effort invested into various malaria elimination programmes and eradication will go to waste.

## 3. Conclusions

This Special Issue will not be able to provide answers and solutions for all current issues encountered in malaria control and prevention. Nevertheless, each of the works compiled here has contributed knowledge and insights into malaria research and management. Importantly, these works may encourage greater attention to the respective niches of malaria research, potentially resulting in improved strategies against the transmission of malaria.
